# Rumen Microbial Diversity and Metabolome Analysis Reveals the Effects of Alkaline Metal Ion Complexes on Muscle Quality of Lambs

**DOI:** 10.3390/biology14121791

**Published:** 2025-12-16

**Authors:** Yang Zi, Yilin Yang, Mingyue Li, Yalin Li, Ziyi An, Mengjiao Liu, Chi Ma, Feng Gao, Changqing Li

**Affiliations:** 1 College of Animal Science, Inner Mongolia Agricultural University, Hohhot 010018, China; zy252270@163.com (Y.Z.); y905963976@163.com (Y.Y.); yue28256@163.com (M.L.); liyalin827@163.com (Y.L.); necvk25@163.com (Z.A.); xiaoxuhao0923@163.com (M.L.); mc1994@emails.imau.edu.cn (C.M.); 2 Institute of Animal Nutrition and Feed, Inner Mongolia Academy of Agricultural and Animal Husbandry Science, Hohhot 010031, China

**Keywords:** alkaline metal ion complex, lamb, rumen microbiota, metabolism, meat quality

## Abstract

This study investigated the effects of dietary supplementation with an alkaline metal ion complex (AMIC) on growth performance, meat quality, rumen microbiota, and metabolome in Hu lambs. Fifty lambs were randomly assigned to either a control group (basal diet) or an AMIC group (basal diet + 0.15% AMIC) for 60 days. The results showed that AMIC significantly increased carcass weight, *Longissimus dorsi* area, crude protein, intramuscular fat, ash content, and meat luminosity (L*). Amino acid profiles and key flavor compounds were elevated, while off-flavor hydrocarbons were reduced. 16S rRNA sequencing revealed that AMIC altered rumen microbiota composition, enriching butyrate-producing genera such as Butyrivibrio and Saccharofermentans. Metabolomic analysis identified 398 differentially expressed metabolites, with upregulated pathways including butanoate metabolism and xylene degradation. Correlation analyses indicated strong associations between specific microbial taxa, metabolites, and meat quality traits. These findings suggest that AMIC enhances meat quality by modulating rumen microbial ecology and metabolic pathways, leading to improved nutrient deposition and flavor development. This study provides novel insights into the microbe–metabolite–muscle axis in ruminants and supports the use of AMIC as a dietary strategy for quality lamb production.

## Simple Summary

Alkaline metal ion complexes play crucial roles in biological systems. Lamb meat, as a high-value food product, makes research into enhancing its quality particularly significant. This study investigated the effects of supplementing Hu lamb diets with an alkaline mineral ion complex. We evaluated growth performance, slaughter traits, and apparent meat quality parameters. These assessments were combined with microbiome and metabolome analyses to elucidate the mechanisms by which alkaline mineral ions influence lamb meat quality. Our findings demonstrate that dietary supplementation with the alkaline mineral ion complex enhances lamb growth performance, modulates fatty acid and amino acid profiles in the meat, and directly improves key meat quality attributes including color and tenderness. Furthermore, it indirectly enhances the flavor profile and overall meat quality through modifications in the gastrointestinal microbiome and metabolome of the lambs.

## 1. Introduction

Mutton is highly regarded by consumers due to its low cholesterol level, high nutritional value, and desirable sensory attributes, with meat quality determined by the protein/fat composition, color, tenderness, and flavor precursors [1].

It is well established that dietary composition critically influences meat quality in livestock. Specifically, the strategic inclusion of appropriate feed ingredients can enhance key quality attributes such as sensory characteristics, nutritional value, and overall product safety [2,3,4,5]. Dietary interventions, including feed additives targeting mineral balance, are critical modulators of these traits [6]. Alkaline metal ions (e.g., K^+^, Na^+^) serve as enzymatic cofactors and osmoregulators, directly influencing muscle hydration and proteolytic activity [7]. In monogastric species, these ions enhance growth performance by improving intestinal barrier function and microbial diversity [8], while promoting intramuscular fat deposition and antioxidant capacity [9]. Existing studies have demonstrated that dietary supplementation with zinc sources can improve rumen fermentation characteristics and nutrient digestibility [10,11]. However, their role in ruminants remains underexplored, particularly regarding interactions among ionic regulation, microbial ecology, and host metabolic signaling.

We hypothesize that dietary alkaline metal ion complexes (AMICs) synergistically improve lamb meat quality through a tripartite mechanism integrating ionic, microbial, and metabolic pathways. AMIC may stabilize muscle cell ion gradients, preserve myofibrillar integrity, and enhance water-holding capacity [12]. AMIC can also enhance intestinal health by selectively enriching beneficial gut microbiota [13]. Furthermore, through inducing modifications in microbial metabolites, AMIC may activate specific host signaling pathways, thereby directing the targeted accumulation and biosynthesis of amino acids, fatty acids, and flavor precursor compounds [14]. Collectively, these pathways bridge ionic homeostasis, microbial dynamics, and metabolic reprogramming to optimize muscle quality.

Building on this framework, we propose that AMIC supplementation in Hu sheep lambs may reconcile rumen microbial ecology with muscle biochemical networks, ultimately enhancing meat quality traits. We therefore systematically characterize the responses of growth performance, meat quality, and the rumen microbiome and metabolome to AMIC in Hu sheep, elucidating its integrated effects to fill a critical void in the current understanding.

## 2. Materials and Methods

The experimental protocols were approved by the Animal Care and Ethics Committee of Inner Mongolia Agricultural University (approval number NND 2021105). All procedures complied with institutional guidelines for the care and use of laboratory animals in China.

### 2.1. Animals

**Figure 1:**
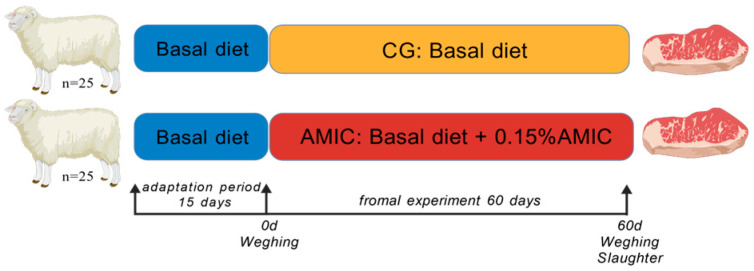
Schematic diagram of the experimental procedure.

A schematic of the experimental procedure used to examine the effects of AMIC supplementation on meat quality of Hu sheep lambs is shown in Figure 1. Fifty male Hu lambs (wethers) approximately three months of age were selected for the study based on strict inclusion criteria. To ensure homogeneity and minimize the confounding effect of initial body weight, lambs with a body weight of 30.0 ± 2.5 kg were chosen. The health status of each lamb was assessed and confirmed by a veterinarian through a standard clinical examination prior to enrollment. This examination verified the absence of any clinical signs of disease, such as respiratory distress (e.g., coughing), digestive disorders (e.g., diarrhea), or lameness. Any animal not meeting all these criteria was excluded prior to the subsequent procedures.

After a 15-day adaptation period, the lambs were randomly allocated to one of two dietary treatments in a pen-based design: the control group (CG, *n* = 25), fed a basal diet, and the AMIC group (AMIC, *n* = 25), supplemented with 0.15% AMIC. At a concentration of 0.15% of the TMR mass (DM basis), AMIC was premixed into the ration to ensure homogeneous distribution before the feeding period. Each group consisted of six pens (replicates), with five pens housing four lambs and one pen housing five lambs. The composition of the basic diet is shown in Table 1, and the alkaline metal ion complex (comprising elements including K^+^, Na^+^, Zn^2+^, and Ge^4+^) was purchased from Beijing Jinnaer Biotechnology Co., Ltd. (Beijing, China). Lambs were raised for 60 days on a total mixed ration (specific composition in Table 2), fed twice daily (07:00 and 19:00). Body weight (BW) was recorded on days 0, 30, and 60, followed by the calculation of average daily gain (ADG) and average daily feed intake (ADFI) for each period. All animals had ad libitum access to water.

### 2.2. Carcass and Meat Quality Evaluation

#### 2.2.1. Determination of Slaughter Performance

At the end of the experimental period, six sheep from each group were randomly selected and humanely slaughtered. Rumen fluid, collected prior to slaughter, was clarified through sequential filtration and immediately snap-frozen in liquid nitrogen. We determined the carcass weight, carcass fat value (GR), backfat thickness, and eye muscle area. The longissimus dorsi muscle was then dissected from one side of the carcass using a surgical scalpel, aliquoted, and similarly snap-frozen. All samples were stored at −80 °C for subsequent analysis.

#### 2.2.2. Determination of pH and Meat Color

Muscle pH was measured 45 min post-slaughter using a pH-STAR probe (Matthäus, Pottmes, Germany), with the pH meter calibrated at room temperature (25 °C) using standard buffers of pH 4.0 and 7.0. Meat color was assessed according to the method of M. P. Ellies-Oury et al. [15]. A chromameter (TC-P2A, Kett, Tokyo, Japan) was used to determine the lightness (L*), redness (a*), and yellowness (b*) at the upper, middle, and lower portions of the muscle. Each reported color and pH value represents the average of three measurements.

#### 2.2.3. Determination of the Shear Force and the Cooking Percentage

The shear force and cooking percentage were determined according to the method described by He et al. [16]. Identical longissimus dorsi portions from the experimental sheep were aged at 4 °C for 24 h. After removing the fascia and fat, the samples were cut into approximately 3 cm cubes, and a thermometer was inserted into the center of each. These cubes were sealed in bags with the opening facing upward and heated in an 80 °C water bath for 30 min, or until the internal temperature reached 70 °C. The samples were then removed, surface moisture was blotted with filter paper, and they were cooled to room temperature. Cylindrical cores were subsequently extracted using a circular sampler. Shear force was measured with a C-LM digital muscle tenderness meter (C-LM4, YuanBao, Beijing, China), applying a 1000 N compression load cell to cores 1.25 cm in diameter. Four replicates per sample were analyzed to ensure reliable shear force determination. For cooking percentage, identical muscle portions were weighed accurately (±0.01 g) to obtain mass m_1_. Each sample was sealed in a cooking bag after air expulsion and fully immersed in an 80 °C thermostatic water bath (SHHW21, YueJin, Beijing, China) for 35 min. Following heating, the bags were cooled under running cold water for 30 min. The samples were then removed, surface moisture was blotted, and they were reweighed to obtain mass m_2_. All samples were processed in a single batch under identical conditions. Cooking percentage was calculated as (m_2_/m_1_) × 100.

#### 2.2.4. Determination of Nutritional Components in Meat

Moisture, ash, crude protein, and crude fat contents were determined according to the method described by Zhang et al. [17]. Muscle moisture content was measured using a freeze-dryer (EYEKA, FDU-2110; Tokyo Rikakikai Co., Ltd., Tokyo, Japan) until a constant weight was achieved. Ash content was determined through muffle furnace combustion, crude protein through the Kjeldahl method, and crude fat through Soxhlet extraction, with each sample analyzed in triplicate.

### 2.3. Amino Acids and Fatty Acids

Amino acids were quantified using a Waters ACQUITY UPLC I-Class system coupled to an XEVO TQ-S Micro Series mass spectrometer (Waters, Shanghai, China). Approximately 50 mg of muscle tissue was homogenized in 50% methanol/water, vortexed, and centrifuged. The resulting supernatant was then derivatized with AccQ·Tag™ reagent. Chromatographic separation employed a UPLC HSS T3 column with gradient elution, and detection was performed in ESI+ mode. We quantified the amino acids based on external standard curves (1–400 μmol/L) using MassLynx™ software v4.1, with each sample analyzed in technical duplicate.

According to the method described by Al-Soufi et al. [18], we analyzed the fatty acid composition as fatty acid methyl esters (FAMEs) using gas chromatography–mass spectrometry (GC-MS; Agilent 8860/5977C, Santa Clara, CA, USA). Separation was achieved on an Agilent DB-FastFAME column with a specific temperature program. Quantification relied on external standard curves using Agilent MassHunter software 5.4, and we calculated various nutritional indices from the resultant data.

### 2.4. Flavor Substances

The 2-methyl-3-heptanone standard was added to the muscle samples, and volatile flavor compounds were analyzed through gas chromatography–mass spectrometry (GC-MS, 7890B/5977A, Agilent, USA) using a sample-specific temperature program. Quantification of these compounds was achieved by applying the established linear relationship between the peak area and concentration of the 2-methyl-3-heptanone standard. Each sample underwent a single GC-MS analysis in full scan mode to acquire spectral data for both compound identification and quantification.

### 2.5. Rumen Microbiota Diversity and Abundance Analysis

#### 2.5.1. High-Throughput 16S Ribosomal RNA Gene Sequencing

Total genomic DNA was extracted from samples using the TGuide S96 Magnetic Soil/Stool DNA Kit (Tiangen Biotech, Beijing, China). Following extraction, the hypervariable V3-V4 region of the bacterial 16S rRNA gene was amplified with primers 338F (5′-ACTCCTACGGGAGGCAGCA-3′) and 806R (5′-GGACTACHVGGGTWTCTAAT-3′) [19]. PCR products were checked on agarose gel and purified through the Omega DNA purification kit (Omega Inc., Norcross, GA, USA). The purified PCR products were collected, and the paired ends (2 × 250 bp) were determined with the Illumina Novaseq 6000 platform (San Diego, CA, USA). Both forward and reverse primers were appended with sample-specific Illumina index sequences to enable multiplexed deep sequencing.

#### 2.5.2. Rumen Microbiota Diversity and Abundance Analysis

The analysis of rumen microbial diversity and abundance followed the protocol established by Shen et al. [20]. Paired-end reads were quality-filtered, merged, and filtered by length. The sequences were taxonomically classified, and operational taxonomic units (OTUs) were defined, filtered, and normalized to relative abundance. Microbial community diversity was analyzed. For predominant clades, sequences were aligned to construct a maximum likelihood phylogenetic tree. Changes in the rumen flora of Hu lambs were analyzed via 16S rRNA sequencing. Valid sequences were clustered at 97% similarity using USEARCH (version 10.0), applying a filtering threshold of 0.005% of all sequenced reads (BMKCloud, Biomarker Technologies Co., Ltd., Beijing, China). A Venn diagram was generated for OTU clustering analysis.

### 2.6. Rumen Metabolomics Analysis

#### 2.6.1. Metabolite Extraction and LC-MS/MS Analysis

Metabolite extraction and LC-MS/MS analysis were performed following a modified protocol from Cho et al. [21]. A Waters Acquity I-Class PLUS UPLC system coupled to a Waters Xevo G2-XS QTOF high-resolution mass spectrometer (Thermo Fisher, Shanghai, China) was used for the analysis, with separation achieved on a Waters Acquity UPLC HSS T3 column (1.8 μm, 2.1 × 100 mm). The mobile phase comprised 0.1% formic acid in water (A) and 0.1% formic acid in acetonitrile (B), with this composition applied in both positive and negative ionization modes. The instrument operated in MSe mode, simultaneously collecting low- and high-energy spectra (ramped from 10 to 40 eV) at a scan rate of 0.2 s per spectrum. ESI source parameters were set as follows: capillary voltage at ±2.5 kV (positive)/−2.0 kV (negative); cone voltage 30 V; source temperature 100 °C; desolvation temperature 500 °C; desolvation gas flow 800 L/h; and curtain gas flow 50 L/h.

#### 2.6.2. Data Preprocessing and Annotation

Raw data were processed using Progenesis QI software V3.0 for peak picking, alignment, and normalization to total ion intensity. Metabolites were annotated by querying the METLIN database and an in-house library, with a mass accuracy tolerance of <10 ppm and a matching of theoretical fragments.

#### 2.6.3. Bioinformatics Analysis

Normalized data were analyzed using principal component analysis and Spearman correlation on QC samples to assess repeatability. Differentially abundant metabolites between the CG and AMIC groups were identified via OPLS-DA (validated by 200 permutation tests) with thresholds of VIP > 1, fold change >1.5 or <0.67, and *p*-value < 0.05. Metabolic pathways were annotated using KEGG, HMDB, and LipidMaps, and enrichment analysis was performed via hypergeometric testing to identify significantly impacted pathways.

### 2.7. Correlation Analyses

To evaluate the associations between the gut microbiome, metabolomic features, and meat quality phenotypes, we conducted Pearson correlation analyses on the following, respectively: differential microbial genera and amino acid content; differential microbial genera and fatty acid content; differential microbial genera and flavor compound content; as well as differential metabolites and amino acid content. We calculated Pearson correlation coefficients and their raw *p*-values one by one between preprocessed genus-level microbial relative abundance features, metabolite concentration features, and normalized phenotypes. We subjected raw *p*-values to multiple testing correction to eliminate false positives. Finally, we visualized the corrected significant correlation results using heatmaps. In these heatmaps, red represents positive correlations and blue represents negative correlations, with darker colors indicating a stronger correlation intensity. Significance levels are marked with asterisks: * denotes significant correlations (*p* < 0.05) and ** denotes highly significant correlations (*p* < 0.01).

### 2.8. Statistical Analysis

Data collection was randomized for all experiments. Non-omics data processing was performed using 10.0.2 GraphPad Prism software (GraphPad; San Diego, CA, USA). All data are expressed as the means ± standard error of the mean (SEM). For assays with technical replicates (e.g., meat color, shear force), the average value for each biological sample was calculated first and used for subsequent statistical analysis. The Shapiro–Wilk test was applied to determine the normality of data distribution in each group, and the Kolmogorov–Smirnov test was also used for this purpose. The F test served to conduct the Homogeneity of Variance Test, examining whether variances are equal. For normally distributed data with unequal variances, the Welch *t* test was adopted. We used the two-tailed unpaired Student’s *t*-test for normally distributed data and the Mann–Whitney U test for non-normally distributed data, with a *p* value < 0.05 considered statistically significant.

## 3. Results

### 3.1. Growth Performance, Carcass, and Meat Quality Evaluation

**Figure 2:**
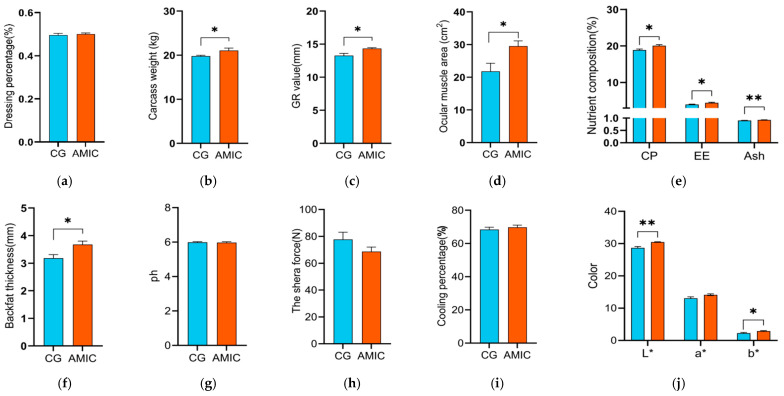
Effect of alkaline metal ion complexes on the slaughter performance and the physicochemical properties in Hu lambs. (**a**) The dressing percentage, %. (**b**) The carcass weight, kg. (**c**) The GR value, mm. (**d**) The ocular muscle area, cm^2^. (**e**) The nutrient composition of longest dorsal muscle of Hu sheep, %. (**f**) The backfat thickness, mm. (**g**) The muscle pH. (**h**) The shear force of muscle, N. (**i**) The cooking percentage of muscle, %. (**j**) The muscle color. The data are presented as the mean ± standard error. Significant differences between the CG and AMIC group, * *p* < 0.05, ** *p* < 0.01. GR, carcass fat. L*, luminosity; a*, intensity of the color red; b*, intensity of the color yellow; CP, crude protein; EE, crude fat; Ash, crude ash.

The effects of AMIC supplementation on the feed intake and growth performance of sheep are presented in Table 3. Compared with the control group, the weight gain and ADG in the AMIC group were significantly higher (*p* < 0.01). The ADFI in the AMIC group was also significantly increased compared to the control group. During the early experimental period, the feed-to-weight gain ratio in the AMIC group was significantly lower than that of the control group (*p* < 0.05), while, over the entire experimental period, a decreasing trend was observed, though it was not statistically significant. The effects of AMIC supplementation on sheep meat quality are shown in Figure 2. Compared with the control group, AMIC lambs showed a significantly greater carcass weight, GR value, ocular muscle area, backfat thickness, yellowness, CP, and EE (*p* < 0.05). Moreover, the ash content and luminosity were significantly higher in the AMIC group (*p* < 0.01). There were no significant differences in the dressing percentage, pH, shear force, cooling percentage, and redness (*p* > 0.05) between the CG group and the AMIC group.

### 3.2. Amino Acids and Fatty Acids in Muscles

**Figure 3:**
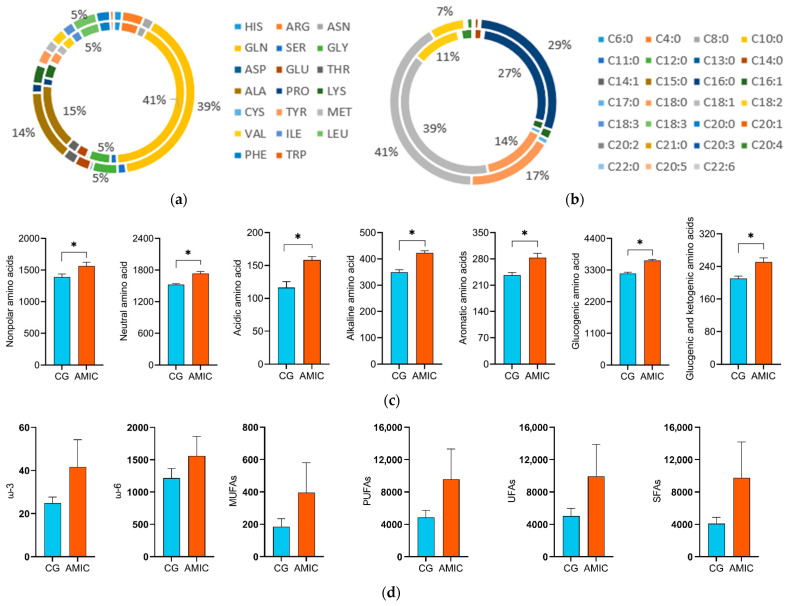
Effect of alkali metal ion complexes on the fatty acids and amino acids in muscle of Hu lambs. (**a**) Effect of alkali metal ion complexes on the amino acids (μg/g). (**b**) Effect of alkali metal ion complexes on the fatty acids (mg/kg). (**c**) Nonpolar amino acids, neutral amino acids, acidic amino acids, alkaline amino acids, aromatic amino acids, glucogenic amino acids, glucogenic and ketogenic amino acids. (**d**) ω-3, ω-6, MUFAs, PUFAs, UFAs, and SFAs. The data are presented as the mean ± standard error. Significant differences between the CG and AMIC group, * *p* < 0.05, CG, the control group; AMIC, the alkali metal ion complex group; SFAs, saturated fatty acids; MUFAs, monounsaturated fatty acids; UFAs, unsaturated fatty acids; PUFAs, polyunsaturated fatty acids.

The amino acid and fatty acid composition in muscles was shown in Figure 3a,b. Among the amino acids, GLN was the most abundant in both groups, constituting 41% in the control group and 39% in the AMIC group. This was followed by ALA, which accounted for 15% in the control group and 14% in the AMIC group. Regarding fatty acids, the predominant compounds in both groups were C18:1 (39% vs. 41%), C16:0 (27% vs. 29%), C18:0 (14% vs. 17%), and C18:2 (11% vs. 7%) in the control and AMIC groups, respectively.

Compared to the controls, the nonpolar amino acids, neutral amino acids, acidic amino acids, alkaline amino acids, aromatic amino acids, glucogenic amino acids, and glucogenic and ketogenic amino acids were significantly increased in the AMIC group (*p* < 0.05) (Figure 3c) (nonpolar amino acids: Gly, Ala, Val, Leu, Ile, Pro, Met, Phe, and Trp; neutral amino acids: Ser, Thr, Cys, Asn, Gln, and Tyr; acidic amino acids: Asp and Glu; alkaline amino acids: Lys, Arg, and His; aromatic amino acids: Phe, Tyr, and Trp; glucogenic amino acids: Gly, Ala, Ser, Thr, Cys, Asn, Asp, Glu, Gln, Pro, Val, Met, Arg, and His; glucogenic and ketogenic amino acids: Ile, Phe, Tyr, and Trp). The contents of SFA, ω-3, ω-6, MUFA, UFA, and PUFA in the AMIC group were increased compared with those of the CG, but not significantly (*p* > 0.05) (Figure 3d).

### 3.3. Flavor Substances

**Figure 4:**
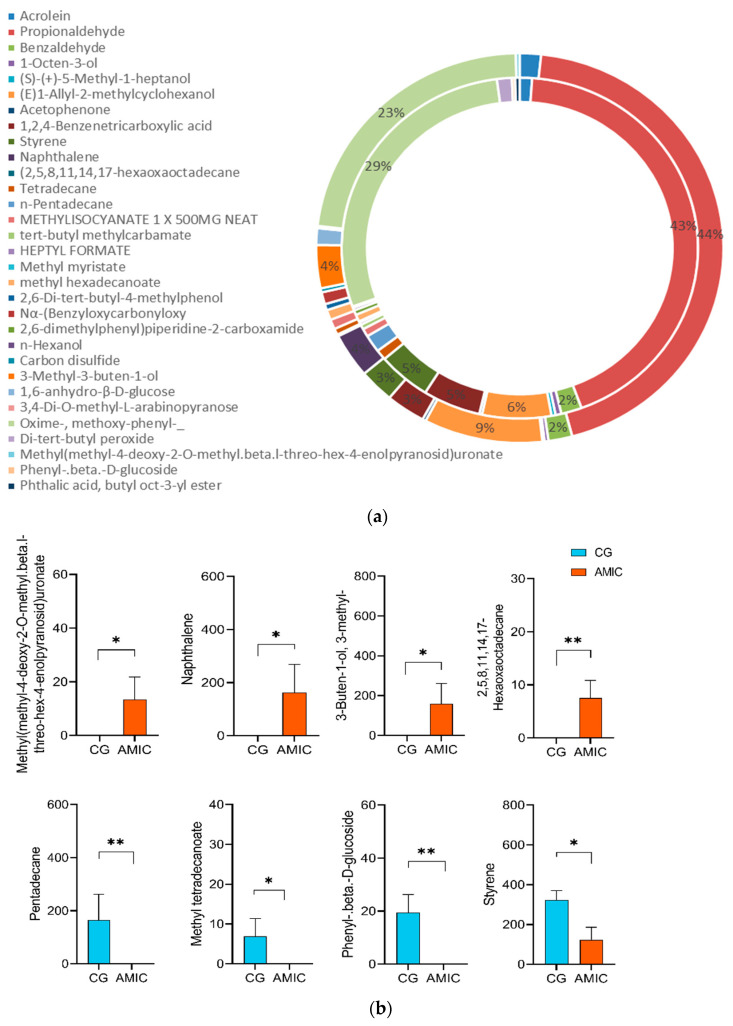
Effects of AMIC on volatile flavor substances in muscle of Hu lambs. (**a**) Composition of volatile flavor substances. (**b**) The flavor substance that exhibits remarkable properties (μg/kg). The data are presented as the mean ± standard error. Significant differences between the CG and AMIC group, * *p* < 0.05, ** *p* < 0.01.

The effect of AMIC on the types of volatile flavor substances in the muscle of Hu lambs is presented in Figure 4. In total, 49 and 46 flavor substances were detected in the control group and AMIC group, respectively. Propionaldehyde was the predominant compound (43% in CG vs. 44% in AMIC), followed by Oxime-, methoxy-phenyl- (29% in CG vs. 23% in AMIC). Relative to the controls, the AMIC group exhibited elevated muscle contents of *2,5,8,11,14,17-Hexaoxaoctadecane*, *Naphthalene*, *Methyluronate*, and *3-Methyl-3-buten-1-ol* (*p* < 0.05). Conversely, the contents of *methyl tetradecanoate*, *pentadecane*, *Phenyl-β-D-glucoside*, and *styrene* were significantly reduced (*p* < 0.05).

### 3.4. Rumen Microbiota Diversity and Abundance

**Figure 5:**
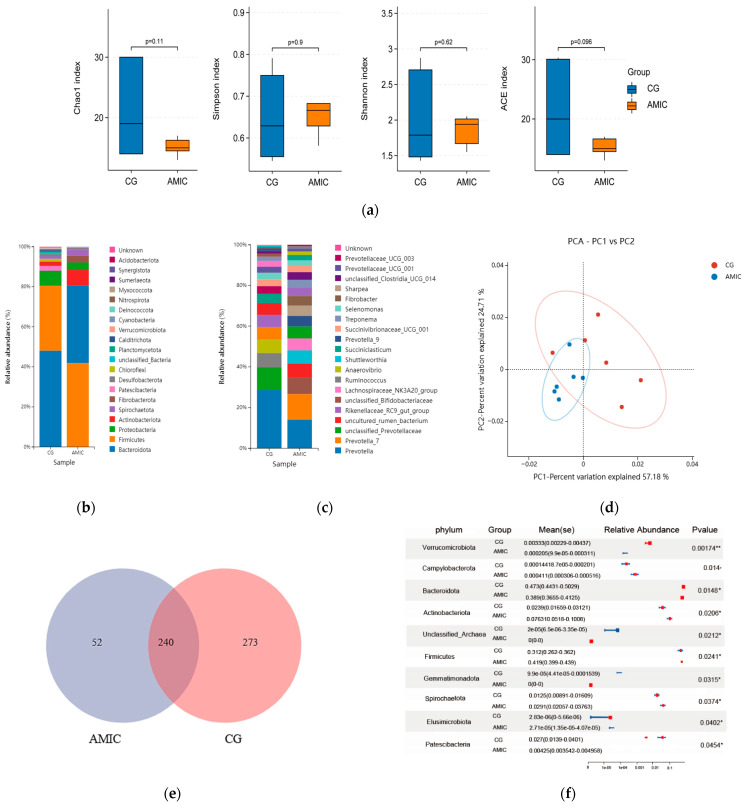
Effects of AMIC on rumen microbiota diversity of Hu lambs. (**a**) Alpha diversity indices at the genus level (Shannon index, Simpson index, Ace index, and Chao index). (**b**) Relative abundance of rumen bacteria at the phylum level. (**c**) Relative abundance of rumen bacteria at the genus level. (**d**) Principal component analysis (PCA). (**e**) Venn diagram at the genus level. (**f**) The significance of differences among the two groups of the same species at the genus level (* represents *p* < 0.05, ** *p* < 0.01; se represents standard error).

**Figure 6:**
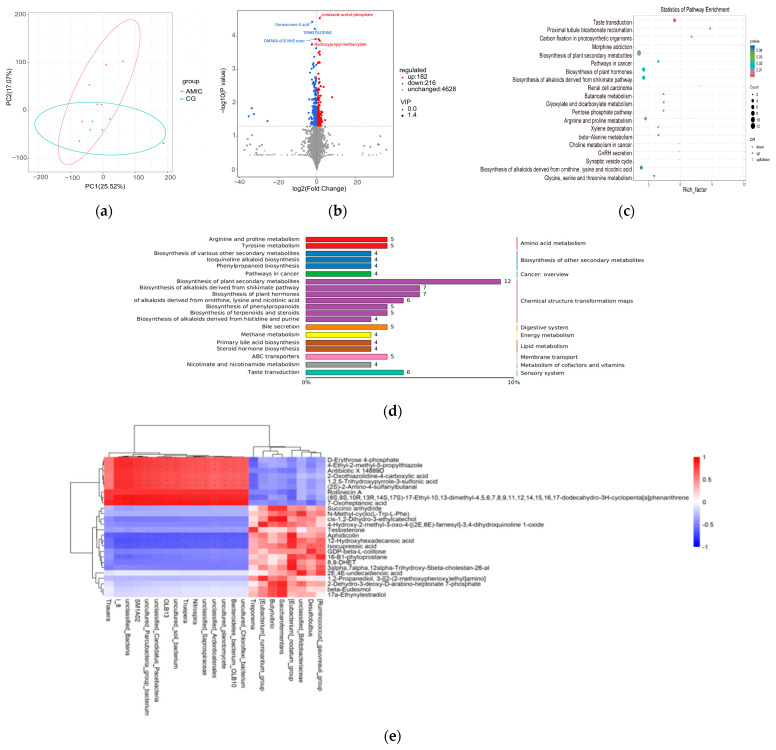
Effect of AMIC on the metabolites in rumen of Hu lambs. (**a**) PLS-DA diagrams. Different colors represent different groups. (**b**) Volcano plot indicates differential metabolites. (**c**) KEGG enrichment analysis of differential metabolites. The bubble plot depicts the enrichment analysis results, plotting pathway names against their rich factor. Dot color represents the enrichment significance (redder hues indicate greater significance), and dot size corresponds to the count of enriched differential metabolites. (**d**) Summary map of the top 20 items with the most different metabolites. (**e**) Correlation heatmap of rumen microbiota and rumen metabolites of Hu lambs. Correlation heatmap: * *p* < 0.05, ** *p* < 0.01.

Although no differences in alpha diversity indices were observed (Figure 5a), AMIC supplementation significantly altered the relative abundance of specific phyla (e.g., Verrucomicrobiota, Campylobacterota) and genera (e.g., Saccharofermentans, Butyrivibrio) (Figure 5b–f). At the phylum level, Bacteroidota and Firmicutes were the dominant gates for the control and AMIC group (Figure 5b). At the genus level, Prevotella, unclassified_Prevotella, and Ruminococcus were the top three dominant genera in the control group (Figure 5c). Prevotella, Prevotella_7, and unclassified_Bifidobacteriaceae were the top three dominant genera in the AMIC group. The results indicated that 57.18% of the variation was captured by axis 1, while axis 2 represents 24.71% of the variation (Figure 5d). As depicted in Figure 5e, the control group contained 273 species at the genus level, while the AMIC group had 52 species at the genus level, and 240 species were found to co-occur across both groups. Compared with the CG group, AMIC increased the relative abundance of Verrucomicrobiota, Campylobacterota, Bacteroidota, Actinobacteriota, unclassified Archaea, Firmicutes, Gemmatimonadota, Spirochaetota, Elusimicrobiota, and Patescibacteria (*p* < 0.05. Figure 5f). These changes were associated with shifts in rumen metabolites (Figure 6), though causality requires further validation.

### 3.5. Rumen Metabolome

The rumen metabolism analysis of Hu lambs is shown in Figure 6. The partial least squares discriminant analysis (PLS-DA) model showed significant differences between the CG group and the AMIC group (*p* < 0.05; Figure 6a). Between the CG and AMIC groups, 398 metabolites were found to be differentially expressed, with 216 downregulated and 182 upregulated (*p* < 0.05; Figure 6b). Metabolic pathways with the most differentiated metabolites were identified, among which the top 20 significant pathways are displayed in Figure 6c,d. These pathways include taste transduction, proximal tubule bicarbonate reclamation, carbon fixation in photosynthetic organisms, morphine addiction, the biosynthesis of plant secondary metabolites, pathways in cancer, the biosynthesis of plant hormones, the biosynthesis of alkaloids derived from the shikimate pathway, renal cell carcinoma, butanoate metabolism, glyoxylate and dicarboxylate metabolism, the pentose phosphate pathway, arginine and proline metabolism, xylene degradation, beta-alanine metabolism, choline metabolism in cancer, GnRH secretion, the synaptic vesicle cycle, the biosynthesis of alkaloids derived from omithine, lysine, and nicotinic acid glycine, and serine and threonine metabolism. Several metabolites were upregulated in three key pathways: butanoate metabolism, xylene degradation, and cancer-related pathways. Several metabolites were downregulated in two key pathways: the pentose phosphate pathway and glycine, serine, and threonine metabolism. Amino acid metabolism contains two paths: arginine and proline metabolism and tyrosine metabolism. The biosynthesis of other secondary metabolites contains three channels: the biosynthesis of various other secondary metabolites, Isoquinoline alkaloid biosynthesis, and phenylpropanoid biosynthesis. The cancer overview contains pathways for choline metabolism in cancer. The chemical structure transformation maps contain the biosynthesis of plant secondary metabolites, the biosynthesis of alkaloids derived from the shikimate pathway, the biosynthesis of plant hormones, the biosynthesis of alkaloids derived from ornithine, lysine, and nicotinic acid, the biosynthesis of phenylpropanoids, the biosynthesis of terpenoids and steroids, and the biosynthesis of alkaloids derived from histidine and purine. The digestive system contains bile secretion. Energy metabolism contains methane metabolism. Lipid metabolism contains primary bile acid biosynthesis and steroid hormone biosynthesis. Membrane transport contains ABC transporters. The metabolism of cofactors and vitamins contains nicotinate and nicotinamide metabolism.

A Spearman correlation analysis was conducted to assess the relationship between the bacterial genera and the rumen metabolite of lambs (Figure 6e). We observed a positive correlation between the variables uncultured_Chloroflexi bacterium, Bacteroidetes_bacterium_OLB10, uncultured planctomycete, unclassified_Saprospiraceae, Nitrospira, uncultured_soil_bacterium, and the compounds D-Erythrose 4-phosphate, 4-ethyl-2-metnyl-5-propyithiazole, Antibiotic X 14889D, 2-Oxothiazolidine-4-carboxylic acid, 1,2,5-Trihydroxypyrole-3-sulfonic acid, (2S)-2-Amino-4-sultanylbutanal, Rolinecin A, and 7-Oxoheptanoic acid (*p* < 0.05). Conversely, a negative correlation was observed between these variables and the compounds Aphidicolin and 12-Hydroxyhexadecanoic acid (*p* < 0.05). Thauera was positively correlated with Rolinecin A and 7-Oxoheptanoic acid (*p* < 0.05), while Thauera was negatively correlated with Aphidicolin (*p* < 0.05). The relationship between the [Eubacterium]_nodatum_group and Rolinecin A was found to be negatively correlated (*p* < 0.05). Similarly, Desulfobulbus exhibited a negative association with 7-Oxoheptanoic acid (*p* < 0.05). The [Ruminococcus]_gauvreauii group showed a positive correlation with several analytes, including 12-Hydroxyhexadecanoic acid, GDP-beta-L-colitose, 16-B1-phytoprostane, 8,9-DHET, 3alpha,7alpha,12alpha-Trihydroxy-5beta-cholestan-26-al, and 2E, 4E-undecadienoic acid (*p* < 0.05). Desulfobulbus was also positively correlated with GDP-beta-L-colitose (*p* < 0.05). There were significant positive correlations between the abundance of the unclassified_Bifidobacteriaceae and N-Methyl-cyclo(L-Trp-L-Phe), 12-Hydroxyhexadecanoic acid, 16-B1-phytoprostane, 2E, 4E-undecadienoic acid, and 2-Dehydro-3-deoxy-D-arabino-heptonate 7-phósphate (*p* < 0.05). The abundance of the [Eubacterium]_nodatum_group was positively correlated with Aphidicolin, 12-Hydroxyhexadecanoic acid, 16-B1-phytoprostane, 8,9-DHET, and 3alpha,7alpha,12aipha-Trihydroxy-5beta-cholestan-26-al (*p* < 0.05). Additionally, Saccharofermentans and Butyrivibrio were positively correlated with Succinic anhydride, cis-1,2-Dinydro-3-ethylcatechol, 2-Dehydro-3-deoxy-D-arabino-heptonate 7-phósphate, beta-Eudesmol, and 17a-Ethynylestradiol (*p* < 0.05). Notably, Saccharofermentans was also positively correlated with N-Methyl-cyclo(L-Trp-L-Phe),1,2-Propanediol (*p* < 0.05). Lastly, the [Eubacterium]_ruminantium_group was positively correlated with 1,2-Propanediol (*p* < 0.05).

### 3.6. Correlation Analysis

**Figure 7:**
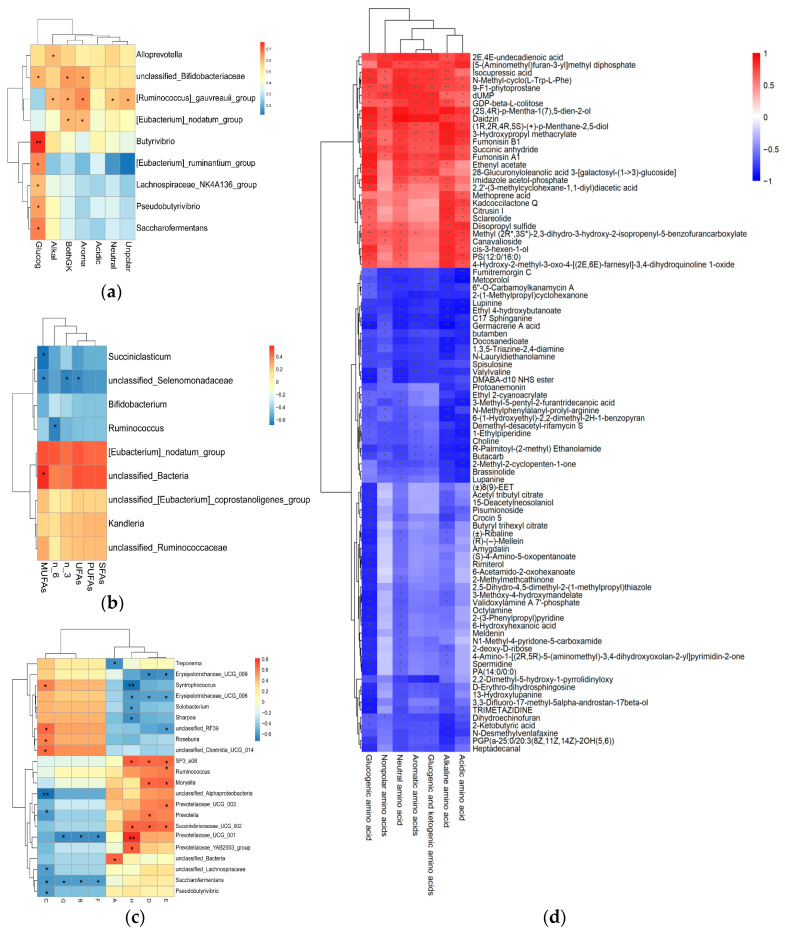
Heat map of correlations between rumen microbiota and amino acids, fatty acids, and flavoring substances in muscle of Hu lambs. (**a**) Correlation heatmap of rumen microbiota and amino acids in muscle of Hu lambs. (**b**) Correlation heatmap of rumen microbiota and fatty acids in muscle of Hu lambs. (**c**) Correlation heatmap of rumen microbiota and flavoring substances in muscle of Hu lambs. (**d**) Correlation heatmap of rumen metabolome and amino acids in muscle of Hu lambs. Note: Correlation heatmap: * *p* < 0.05, ** *p* < 0.01.

A Spearman correlation analysis was performed to assess the relationship between bacterial genera, amino acids, and fatty acids in the muscles of Hu lambs (Figure 7a,b). Glucogenic amino acids were positively correlated with the *[Eubacterium]_ruminantium_group*, *lachnospiraceae_NK4A136_group*, *saccharofermentans*, *pseudobutyrivibrio, butyrivibrio,* and *unclassified_bifidobacteriaceae* (*p* < 0.05). The *[Ruminococcus]_gauvreauii_group* was positively correlated with neutra, unpolar, aroma, bothGK, and alkal (*p* < 0.05). *Unclassified_bifidobacteriaceae* and the *[eubacterium]_nodatum_group* were positively correlated with Aroma and BothGK (*p* < 0.05). *Alloprevotella* was positively correlated with alkal (*p* < 0.05). *N-6 fatty* acid was negatively correlated with *ruminococcus* (*p* < 0.05). MUFAs were negatively correlated with *succiniclasticum* and *unclassified_Selenomonadaceae* (*p* < 0.05) and positively correlated with *unclassified_Bacteria* (*p* < 0.05). *Unclassified_selenomonadaceae* was negatively correlated with N-3 and UFAs (*p* < 0.05).

A Spearman correlation analysis was conducted to assess the relationship between the bacterial genera and the flavor substances in the muscles of Hu lambs (Figure 7c). *2,5,8,11,14,17-Hexaoxaoctadecane* was positively correlated with *syntrophococcus*, *roseburia*, and *unclassified-clostridia-UCG-014* (*p* < 0.05), while *2,5,8,11,14,17-Hexaoxaoctadecane* was negatively correlated with *prevotella, unclassified_lachnospiraceae, pseudobutyrivibrio, saccharofermentans,* and *unclassified-alphaproteobacteria* (*p* < 0.01). *Saccharofermentansb* and *prevotellaceae-UCG-001* were found to exhibit statistically significant negative correlations with *Naphthalene*, *3-buten-1-ol*, *3-methyl-*, and *methyl(methyl-4-deoxy-2-O-methyl.beta.l-threo-hex-4-enolpyranosid)uronate*, respectively (*p* < 0.05), and *styrene* demonstrated a similar negative correlation with *treponem* (*p* < 0.05). *Phenyl-β-D-glucoside* was noted to have a positive correlation with the genes SP3_e08, Prevotellaceae_UCG_001, *succinivibrionaceae_UCG_002*, and *prevotellaceae_YAB2003_group* (*p* < 0.05), while it exhibited a negative correlation with the genera *solobacterium*, *sharpea*, and *syntrophococcus* (*p* < 0.05). *Pentadecane* and *methyl tetradecanoate* were found to have a positive correlation with *SP3_e08*, *moryella*, and *succinivibrionaceae_UCG_002* (*p* < 0.05). Additionally, pentadecane was positively correlated with Prevotella (*p* < 0.05), and *methyl tetradecanoate* was positively correlated with *prevotellaceae_UCG_003* and *ruminococcus* (*p* < 0.05).

A Spearman correlation analysis was conducted to assess the relationship between the rumen metabolome and amino acids in the muscles of Hu lambs (Figure 7d). *2E, 4E-undecadienoic acid, N-methyl-cyclo(L-Trp-L-Phe), 9-F1-phytoprostane, dUMP, GDP-beta-L-colitose, (2S, 4S)-p-Mentha-1(7),5-dien-2-ol, daidzin, (1R, 2R, 4R, 5S)-(+)-P-menthane-2,5-diol, fumonisin B1, succinic anhydride,* and *fumonisin A1* were positively correlated with glucogenic amino acids, acidic amino acids, neutral amino acids, nonpolar amino acids, aromatic amino acids, glucogenic and ketogenic amino acids, and alkaline amino acids (*p* < 0.05). Ethenyl acetate was positively correlated with glucogenic amino acids, neutral amino acids, aromatic amino acids, glucogenic and ketogenic amino acids, and alkaline amino acid (*p* < 0.05). *Kadcoccilactone Q, citrusin I, sclareolide, diisopropyl sulfide, canavalioside,* and *4-hydroxy-2-methyl-3-oxo-4-[(2E,6E)-farnesyl]-3,4-dihydroquinoline 1-oxide* were positively correlated with glucogenic amino acids, acidic amino acids, and alkaline amino acids (*p* < 0.05). *Fumitremorgin C, metoprolol, lupinine, germacrene A acid, butamben, docosanedioate, spisulosine,* and *Butacarb* were negatively correlated with glucogenic amino acids, acidic amino acids, neutral amino acids, nonpolar amino acids, aromatic amino acids, glucogenic and ketogenic amino acids, and alkaline amino acids (*p* < 0.05). *Amygdalin, meldenin,* and *spermidine* were negatively correlated with glucogenic amino acids (*p* < 0.05). 13-Hydroxylupanine and heptadecanal were negatively correlated with glucogenic amino acids, neutral amino acids, aromatic amino acids, glucogenic and ketogenic amino acids, and alkaline amino acids (*p* < 0.05).

## 4. Discussion

The present study provides evidence that dietary supplementation with AMIC enhances growth performance, carcass traits, and meat quality in Hu lambs. By integrating rumen microbiome and metabolome analyses, we further reveal that these improvements may be driven by a tripartite mechanism encompassing ionic balance, microbial community restructuring, and host metabolic alterations. These findings suggest that AMIC acts not only as a nutritional supplement but also as a potential regulator of the gut–muscle interface in ruminants.

The present study observed that dietary AMIC supplementation increased carcass weight, longissimus dorsi area, CP, and intramuscular fat (IMF) content, which aligns with earlier reports on mineral supplementation in animals [9,22]. The marked increase in ash content further indicates an improved mineral retention, reflecting the enhanced bioavailability of ions supplied by AMIC. Zn^2+^, a key cofactor for enzymes such as carboxypeptidases and glutamate dehydrogenase, likely contributed to these outcomes by promoting protein synthesis and optimizing amino acid metabolism [23], thereby elevating the CP content. Meanwhile, the rise in IMF and meat luminosity (L*) may be linked to the antioxidant capacity of Zn^2+^ [24], which helps mitigate lipid and myoglobin oxidation, thus improving color stability and shelf-life. In addition, the synergistic actions of K^+^ and Na^+^ in regulating osmotic balance and cellular hydration are likely responsible for the observed improvements in water-holding capacity, as supported by the trends in the meat quality metrics [25].

A key outcome of this research is the marked restructuring of the rumen microbial community following the addition of AMIC in diets. Notably, we observed increased abundances of Verrucomicrobiota and Campylobacterota, as well as butyrate-producing genera such as Butyrivibrio and Saccharofermentans. Butyrate serves as an essential microbial metabolite that strengthens intestinal barrier integrity and supports energy homeostasis in the host [26]. The pronounced positive correlations between these butyrogenic taxa and glucogenic amino acids (Figure 7a) point to potential host–microbe interactions wherein microbial-derived butyrate may supply energy precursors [27], supporting host muscle protein and amino acid synthesis. This interpretation is corroborated by our metabolomic findings, which indicated a marked upregulation of the butanoate metabolism pathway (Figure 6c). It is plausible that the ionic milieu induced by AMIC, especially elevated K^+^ and Na^+^, promoted the proliferation of these beneficial microorganisms through modifications in ruminal osmotic conditions and fermentation dynamics.

Metabolomic profiling identified substantial changes in the rumen metabolome, with 398 metabolites exhibiting differential expression. The upregulation of pathways such as butanoate metabolism and xylene degradation, alongside the downregulation of the pentose phosphate pathway, reflects a notable shift in energy metabolism within the rumen. Importantly, several key metabolites were established as plausible mechanistic links between AMIC-induced microbial changes and the observed improvements in meat quality. Importantly, the enrichment of key metabolites within microbiota-dependent pathways (e.g., butanoate metabolism) provides a rational, data-driven link. We hypothesize that these AMIC-altered metabolites function as mechanistic connectors, translating the modified rumen microbial activity into the observed systemic improvement in meat quality.

Notably elevated levels of flavor precursors, including N-methyl-cyclo(L-Trp-L-Phe), a cyclic dipeptide with potential aromatic properties, and succinic anhydride, were detected. Their strong positive correlations with Saccharofermentans and Butyrivibrio (Figure 7c) suggest that these microbial taxa may contribute to the biosynthesis of such compounds, possibly via enzymatic activities such as those mediated by cyclic dipeptide synthases [28]. These metabolites are probable precursors involved in Maillard reaction and esterification processes during cooking, thereby directly enhancing the sensory profile of the meat. These reactions are known to generate critical aroma compounds such as pyrazines and thiols [29], which directly contribute to the enhanced flavor and aroma profile of the cooked meat, as confirmed through sensory analysis [30]. Additionally, the reduction in undesirable hydrocarbons such as pentadecane and styrene is consistent with the proposed antioxidant function of Ge^4+^ within AMIC, which likely attenuates lipid peroxidation [31].The Ge^4+^ has been demonstrated to possess potent antioxidant activity [32] and can further exert anti-inflammatory effects [33], thereby comprehensively improving the sensory attributes of meat.

## 5. Conclusions

**Figure 8:**
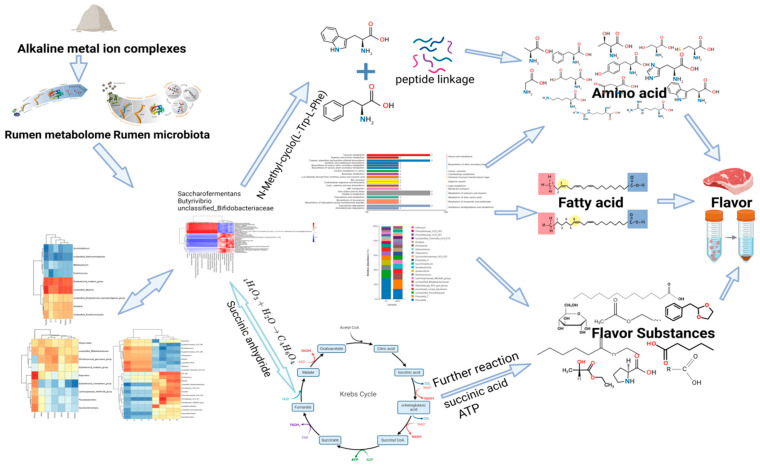
Integrative analysis of the correlation between muscle quality and microbial and metabonomic data of lambs. The complex correlation between different pathways and amino acids, fatty acids, and flavor substances was demonstrated using a multi-group correlation thermogram and network diagram. * *p* < 0.05, ** *p* < 0.01.

In conclusion, our results establish that AMIC supplementation is an effective strategy to enhance the production quality of lamb. We move beyond the conventional nutrient-based explanation and propose a mechanistic model: AMIC modulates the rumen microbiome towards a butyrate-producing phenotype, which in turn reprograms the rumen metabolome to enhance the synthesis of amino acids and favorable flavor precursors. This ultimately translates to an improved muscle nutrient deposition, color, and flavor. This study provides a foundational understanding of the microbe–metabolite–muscle axis in ruminants and highlights the potential of mineral ion-based dietary interventions for precision animal nutrition (Figure 8).

## Figures and Tables

**Table 1:** Ingredient and chemical composition of basal diet (dry matter basis).

Items	Ingredient, %	Nutrient Composition	Content
Corn	33.75	DE ^2^ (MJ/kg)	12.11
Water	16.00	Dry matter (%)	71.13
Corn stalks	13.00	Crude protein (%)	11.76
DDGS ^1^	7.00	Crude fat (%)	1.42
Cotton meal	4.25	Ash	6.39
Soybean meal	4.25	Neutral detergent fiber (%)	38.55
Sprayed corn bran	3.50	Acid detergent fiber (%)	17.23
Rice bran meal	3.25	Calcium (%)	0.58
Corn germ meal	2.50	Phosphor (%)	0.30
Wheat bran	2.50		
Wheat middlings	2.00		
Nutritional feed additives ^3^	2.00		
Jujube powder	1.40		
Fineness stone powder	1.00		
Salt	0.75		
Compound premix feed	0.65		
Urea	0.65		
Soybean germ powder	0.50		
Sodium bicarbonate	0.50		
Probiotic agent ^4^	0.05		
Calcium hydrogen phosphate	0.25		
Calcium sulfate	0.25		
Total	100		

^1^ DDGS: distillers’ dried grains with solubles; ^2^ DE: digestible energy; ^3^ nutritional feed additives: tributyrin (0.1%), cysteamine hydrochloride (0.1%), ammonium chloride (0.4%), rice hull powder (1.4%); ^4^ probiotic agent: yeast culture, 1 × 10^8^ CFU/kg (dry matter basis).

**Table 2:** The concentration of individual elements within the metal ion complex.

Ions	Calculated Concentration, mg/kg
Na^+^	27,482.00
K^+^	25,103.00
Zn^2+^	0.13
Ge^4+^	36,218.00

**Table 3:** Effects of AMIC on feed intake and growth performance in sheep.

Item	CG ^1^	AMIC ^2^	SEM	*p*-Value
WG ^3^ (kg)				
0–30 d	4.78 ^a^	6.12 ^c^	0.31	0.0033
30–60 d	5.40	6.14	0.33	0.1154
0–60 d	10.18 ^a^	12.26 ^c^	0.43	0.0012
ADG ^4^ (g/d)				
0–30 d	159.33 ^a^	204.00 ^c^	10.21	0.0033
30–60 d	180.00	204.67	10.88	0.1154
0–60 d	169.67 ^a^	204.33 ^c^	7.11	0.0012
ADFI ^5^ (kg/d)				
0–30 d	1.17	1.27	0.05	0.1597
30–60 d	1.35 ^a^	1.52 ^c^	0.01	<0.0001
0–60 d	1.26 ^a^	1.39 ^c^	0.03	0.0010
F/G ^6^				
0–30 d	8.47 ^a^	6.58 ^b^	0.57	0.0224
30–60 d	8.14	8.51	0.74	0.7222
0–60 d	7.75	6.99	0.28	0.0596

^1^ AMIC, alkaline metal ion complex group; ^2^ CG, control group; ^3^ WG, weight gain; ^4^ ADG, average daily gain; ^5^ ADFI, average daily feed intake; ^6^ F/G, feed-to-weight gain ratio. Within the same row, means with different superscripts are significantly different (*p* < 0.01).

## Data Availability

The data used to support the results of the current study are available from the corresponding authors upon request.

## Abbreviations

The following abbreviations are used in this manuscript:

AMIC	Alkaline Metal Ion Complexes
GR	Carcass Fat Value
CG	Control Group
BW	Body Weight
ADG	Average Daily Gain
OUT	Operational Taxonomic Unit
ADFI	Average Daily Feed Intake
CP	Crude Protein
EE	Ether Extract
SFA	Saturated Fatty Acids
MUFA	Monounsaturated Fatty Acids
UFA	Unsaturated Fatty Acids
PUFA	Polyunsaturated Fatty Acids
IMF	Intramuscular Fat
